# Broad-spectrum and *Watch* antimicrobials are commonly used to treat hospital-acquired infections in German acute care hospitals: results from the 2022 national point prevalence survey

**DOI:** 10.1186/s13756-025-01608-4

**Published:** 2025-07-17

**Authors:** Seven Johannes Sam Aghdassi, Selin Saydan, Frieder Pfäfflin, Miriam Songa Stegemann, Anja Theloe, Michael Behnke, Luis Alberto Peña Diaz, Alexander Gropmann, Christine Geffers, Brar Piening, Sonja Hansen

**Affiliations:** 1 Charité – Universitätsmedizin Berlin, corporate member of Freie Universität Berlin and Humboldt-Universität zu Berlin, Institute of Hygiene and Environmental Medicine, Berlin, Germany; 2 National Reference Centre for Surveillance of Nosocomial Infections, Berlin, Germany; 3 Charité – Universitätsmedizin Berlin, corporate member of Freie Universität Berlin and Humboldt-Universität zu Berlin, Department of Infectious Diseases and Respiratory Medicine, Berlin, Germany; 4 Charité – Universitätsmedizin Berlin, corporate member of Freie Universität Berlin and Humboldt-Universität zu Berlin, Pharmacy Department, Berlin, Germany

**Keywords:** Antimicrobial use, Antimicrobial stewardship, Surveillance, Point prevalence survey, Infection control

## Abstract

**Background:**

Hospital-acquired infections (HI) and associated antimicrobial use (AU) significantly contribute to antimicrobial resistance. We aimed to analyse AU patterns for HI treatment in German acute care hospitals.

**Methods:**

We analysed data from the German 2022 point prevalence survey (PPS) on AU and healthcare-associated infections, using the European Centre for Disease Prevention and Control protocol across 252 hospitals. Analyses focused on key infection prevention and control (IPC), antimicrobial stewardship (AMS) indicators and AU for HI, categorised by the World Health Organisation AWaRe classification. Comparisons were made to the previous national PPS in 2016 and 2011.

**Results:**

A total of 22 422 antimicrobial prescriptions were recorded in 66 586 patients. HI treatment accounted for 20% of AU. Penicillins with beta-lactamase inhibitors, carbapenems and third-generation cephalosporins accounted for over 50% of AU for HI treatment. *Watch* antimicrobials dominated HI treatment prescriptions, accounting for around 62% of use, particularly in respiratory infections, while use of *Access* antimicrobials was limited (24%). Skin and soft tissue as well as bone and joint infections, respiratory infections, and urinary tract infections were the most commonly treated HI. Over time, IPC indicators, such as alcohol-based hand rub consumption and IPC staffing, improved significantly, yet AMS staffing remained low and comprehensive hospital-wide post-prescription reviews were limited to around a quarter of hospitals.

**Conclusions:**

The findings underscore the importance of reducing HI to curb the use of broad-spectrum antimicrobials in German hospitals. IPC should be included in AMS strategies, alongside aspects like improving AMS staffing and establishing post-prescription review programmes.

## Background

Healthcare-associated infections and their anti-infective treatment are significant contributors to the global rise of antimicrobial resistance (AMR), particularly since these are frequently caused by multidrug-resistant organisms [1–3]. Effective surveillance programmes are essential to monitor trends in healthcare-associated infection prevalence and antimicrobial use (AU), providing a foundation for informed decision-making in both antimicrobial stewardship (AMS) and infection prevention and control (IPC) [4–6]. Point prevalence surveys (PPS) allow for the collection of comprehensive, patient-level data on AU, offering more detailed insights than aggregated consumption data alone [6]. This patient-based data provides the granularity needed to understand antimicrobial prescribing practices in specific clinical contexts, especially for healthcare-associated infections.

Germany has been actively participating in the European PPS on healthcare-associated infections and AU, a project initiated by the European Centre for Disease Prevention and Control (ECDC), with national surveys conducted in 2011, 2016, and 2022 [7–9]. Previous PPS analyses have shown an increasing reliance on broad-spectrum antimicrobials in Germany [9, 10]. Furthermore, it is notable that consistently, approximately one-fifth of all antimicrobial prescriptions recorded in prevalence surveys in German hospitals were for treating hospital-acquired infections (HI) [8, 9]. This substantial proportion of AU associated with HI suggests a significant opportunity to reduce overall AU through targeted IPC measures.

While an overview of AU from the German 2022 PPS has been previously published [9], this article presents a deeper analysis of AU specifically for treating HI, describing the types of antimicrobials prescribed, the indications driving their use, and shifts in prescribing patterns. This investigation will examine AU according to the WHO’s AWaRe (*Access*, *Watch*, *Reserve*) classification [11], utilising a widespread classification system for AU. The findings will seek to inform AMS and IPC activities in German healthcare facilities.

## Methods

The German PPS 2022 was organised by the German National Reference Centre for Surveillance of Nosocomial Infections and followed the standardised PPS protocol developed by the ECDC to assess healthcare-associated infections and AU [6]. The protocol was adapted to the German healthcare setting to ensure applicability across participating hospitals. Hospitals were invited based on stratified sampling from the national hospital registry, ensuring representation across various sizes and levels of care. Furthermore, all hospitals participating in the German national surveillance system KISS (*Krankenhaus-Infektions-Surveillance-System*) were invited to participate. Participation in the PPS was voluntary.

### Data collection

Data collection took place between May and July 2022, employing the ECDC’s “light protocol” to facilitate efficient data capture without compromising details on healthcare-associated infections and AU. Local healthcare personnel received structured training before data collection to ensure adherence to the PPS protocol. Data collectors, primarily from IPC and AMS teams, reviewed each eligible patient’s records, documenting healthcare-associated infection and AU metrics based on ECDC specifications. All data were entered directly into a secure online portal, specifically designed for the PPS project. A final data set, locked on 19 September 2022, included hospitals meeting the minimum requirement of including at least 50% of hospital beds, and excluded those with substantial data inconsistencies. The details of data collection have been described previously [9].

For healthcare-associated infections, data collectors had to apply the ECDC case definitions [6]. For AU, data collectors had to document antimicrobials for systemic use with parenteral, oral, rectal or per inhalation routes of administration. Topical antimicrobials were excluded. Moreover, treatment for tuberculosis was excluded, but drugs for the treatment of tuberculosis were included, if they were used for the treatment of other infections (e.g., mycobacterial infections other than tuberculosis). Moreover, the indication for the prescription as per assessment of the treating physicians was recorded. For instance, for prescriptions that were recorded as “treatment for hospital-acquired infection”, this meant that the treating physician assumed the presence of an HI. However, this assessment was not validated with ECDC case definitions for healthcare-associated infections. Effectively, the data on healthcare-associated infections (as per ECDC case definitions) and AU are not dependant on each other and represent two separate datasets. For the purpose of this analysis, only data derived from the “antimicrobial use part” (i.e., as per treating physician’s assessment) was considered. Antimicrobials with all included routes of administration (see above) were considered. Concerning IPC and AMS key indicators, the following data were considered: annual consumption of alcohol-based hand rub, annual number of blood cultures taken, annual number of stool tests for *Clostridioides difficile* infection, staffing with IPC and AMS personnel, participation in surveillance networks for AMR and antimicrobial consumption, availability of microbiological tests on the weekend, and presence of formal procedure for post-prescription review of antimicrobials. Such a review ought to at least entail broad-spectrum or reserve antimicrobials, and be performed by staff other than the treating physicians within 72 h of prescription [6].

Data collection and analysis were performed in accordance with the legal surveillance requirements specified in the German Infection Protection Act. Therefore, neither ethics committee approval nor informed consent was required.

### Endpoints

The article at hand focused on the following endpoints:

- Assessment of key indicators of IPC and AMS that were recorded
- Describing the proportion of AU for HI treatment and commonly used antimicrobials
- Site-specific HI analysis, identifying the most frequent HI and the associated antimicrobial treatments
- AU categorisation according to the WHO AWaRe classification version 2023 [11]
- Comparative analysis with previous PPS data from 2011 and 2016, assessing IPC and AMS key indicators, as well as trends and changes in prescribing patterns over time

### Data analysis

All recorded antimicrobials were grouped according to the WHO AWaRe classification. Antimicrobials not included in the classification were included in all analyses, but were classified as “not applicable” in the context of AWaRe classification related analyses. A descriptive analysis was performed on data from all hospitals meeting the inclusion criteria. All analyses were conducted using R software (version 4.2.3) and the package epiR (version 2.0.58) was used [12, 13]. Statistical significance was defined with a p-value threshold of 0.05. Chi-square test was used for AU data. Point estimates and confidence intervals were calculated for prescriptions per 100 patients. Wilcoxon rank-sum test and Kruskal–Wallis test were used for process and structure data to test for differences between two and three samples respectively.

## Results

Altogether, 262 hospitals participated in the PPS 2022, of which 10 had to be excluded due to methodological inconsistencies. Accordingly, the final dataset of the PPS 2022 comprised 252 hospitals. Of these, 29 (11.5%) were tertiary care hospitals. About half of hospitals were publicly owned (*n* = 121, 48.0%). The median hospital size was 300 beds, and hospitals reported a median length of stay of 5.7 days. A total of 93 hospitals participated both in the PPS 2016 and PPS 2022, which included 25 hospitals that participated in all three surveys. Table 1 summarises key indicators of IPC and AMS and provides a comparison with the previous PPS in 2016 und 2011.

**Table 1:** Key indicators of infection prevention and control and antimicrobial stewardship. Data from 252 hospitals participating in the point prevalence survey (PPS) 2022, 218 hospitals participating in the PPS 2016 and 132 hospitals participating in the PPS 2011

Parameter	PPS 2022	PPS 2016	PPS 2011	*p*-value*
AHR in mL per patient day (Median (IQR))	41.6 (33.2–57.4)	32.5 (25.0–51.4)	24.5 (17.6–38.1)	< 0.001
Blood cultures per 1000 patient days (Median (IQR))	43.0 (23.4–58.2)	20.9 (12.5–31.3)	N/A	< 0.001
CDI stool tests per 1000 patient days (Median (IQR))	6.1 (4.0–9.0)	7.3 (4.4–11.0)	N/A	0.021
Beds per full-time infection control nurse (Median (IQR))	167 (134–205)	203 (172–257)	354 (278–460)	< 0.001
Beds per full-time infection control physician (Median (IQR))	704 (461–1112)	817 (513–1562)	1570 (852–3663)	< 0.001
Beds per full-time AMS consultant (Median (IQR))	1121 (601–2360)	1525 (714–2140)	N/A	0.481
Full-time AMS consultants per 250 beds (Median (IQR))	0 (0–0.1)	0 (0–0.1)	N/A	0.016
Participation in surveillance network for AMR (Number (Percentage), Number of datasets)	96 (38.1), *n* = 252	56 (25.7), *n* = 218	N/A	0.006
Participation in surveillance network for antimicrobial consumption (Number (Percentage), Number of datasets)	155 (61.5), *n* = 252	83 (38.1), *n* = 218	N/A	< 0.001
Microbiology: Clinical tests available on Saturday (Number (Percentage), Number of datasets)	241 (98.4), *n* = 245	209 (95.9), *n* = 218	N/A	0.180
Microbiology: Clinical tests available on Sunday (Number (Percentage), Number of datasets)	228 (93.4), *n* = 244	170 (80.6), *n* = 211	N/A	< 0.001
Microbiology: Screening tests available on Saturday (Number (Percentage), Number of datasets)	234 (96.3), *n* = 243	207 (95.4), *n* = 217	N/A	0.801
Microbiology: Screening tests available on Sunday (Number (Percentage), Number of datasets)	216 (88.9), *n* = 243	171 (81.8), *n* = 209	N/A	0.045
Formal procedure for post-prescription review in place in all wards (Number (Percentage), Number of datasets)	59 (25.8), *n* = 229	35 (17.5), *n* = 200	N/A	0.051
Formal procedure for post-prescription review in place in selected wards only (Number (Percentage), Number of datasets)	31 (13.5), *n* = 229	22 (11), *n* = 200	N/A	0.516
Formal procedure for post-prescription review in place in ICU only (Number (Percentage), Number of datasets)	35 (15.3), *n* = 229	22 (11), *n* = 200	N/A	0.245
No formal procedure for post-prescription review in place (Number (Percentage), Number of datasets)	104 (45.4), *n* = 229	121 (60.5), *n* = 200	N/A	0.002

*Abbreviations*: *AHR* Alcohol-based hand rub, *AMS* Antimicrobial stewardship, *AMR* Antimicrobial resistance, *CDI Clostridioides difficile* infection, *ICU* Intensive care unit, *IQR* Interquartile range, *mL* millilitre, *N/A* Not applicable, *PPS* Point prevalence survey

^*^p-values were calculated with Wilcoxon rank-sum test for parameters with data from two samples and Kruskal–Wallis test for parameters with data from three samples

Notably, the consumption of alcohol-based hand rub and the frequency of blood culture sampling increased significantly over time. Moreover, staffing with IPC nurses and physicians improved significantly from 2011 to 2022. Conversely, staffing with AMS consultants remained at a low level, with the majority of hospitals employing 0 full-time consultants per 250 hospital beds. Participation in surveillance networks for AMR and antimicrobial consumption was higher in 2022 than 2016. Only a minority of hospitals (59 of 229 respondents, 25.8%) reported having a policy in place in all wards for a post-prescription review of antimicrobials. However, this was substantially higher than in 2016 (17.5%). Correspondingly, the percentage of hospitals with no policy for post-prescription reviews was lower in 2022 than in 2016 (45.4% vs. 60.5%).

In 66 586 patients that were observed in the PPS 2022, a total of 22 422 antimicrobial prescriptions were recorded, resulting in a prevalence of patients with at least one antimicrobial of 26.9%. When differentiating antimicrobial prescriptions by indication, 4 367 (19.5%) prescriptions were for the treatment of HI. This was consistent over time, with similar values in 2016 (4 411 of 22 086, 20.0%) and 2011 (2 670 of 14 076, 19.0%). Broad-spectrum agents, such as penicillins with beta-lactamase inhibitors, carbapenems and third-generation cephalosporins represented the most frequently administered antimicrobials for HI in the PPS 2022, accounting for over 50% of all antimicrobials for that indication (Table 2).

**Table 2:** The ten most frequently prescribed antimicrobial groups for the treatment of hospital-acquired infections. Data from 252 hospitals participating in the point prevalence survey (PPS) 2022, 218 hospitals participating in the PPS 2016 and 132 hospitals participating in the PPS 2011

Antimicrobial group	Number of prescriptions (PPS 2022)	Proportion* (%) (PPS 2022)	Point estimate of prescriptions per 100 patients (95% CI) (PPS 2022)	Point estimate of prescriptions per 100 patients (95% CI) (PPS 2016)	Point estimate of prescriptions per 100 patients (95% CI) (PPS 2011)	*p*-value**
Penicillins with beta-lactamase inhibitors	1383	31.7	2.1 (2.0–2.2)	1.5 (1.4–1.6)	0.8 (0.7–0.9)	< 0.001
Carbapenems	584	13.4	0.9 (0.8–1)	0.8 (0.7–0.9)	0.7 (0.6–0.8)	0.011
Third-generation cephalosporins	307	7.0	0.5 (0.4–0.5)	0.4 (0.3–0.4)	0.5 (0.5–0.6)	0.008
Other antibacterials (J01XX)	273	6.3	0.4 (0.4–0.5)	0.3 (0.2–0.3)	0.2 (0.2–0.3)	< 0.001
Glycopeptides antibacterials	253	5.8	0.4 (0.3–0.4)	0.4 (0.4–0.5)	0.5 (0.4–0.6)	0.023
Fluoroquinolones	222	5.1	0.3 (0.3–0.4)	0.9 (0.8–1.0)	1.0 (0.9–1.1)	< 0.001
Penicillins with extended spectrum	154	3.5	0.2 (0.2–0.3)	0.1 (0.1–0.1)	0.2 (0.2–0.3)	< 0.001
Antibiotics (A07AA)	132	3.0	0.2 (0.2–0.2)	0.3 (0.2–0.3)	0.2 (0.1–0.2)	0.005
Beta-lactamase-resistant penicillins	124	2.8	0.2 (0.2–0.2)	0.1 (0.1–0.1)	0.1 (0.0–0.1)	< 0.001
Second-generation cephalosporins	114	2.6	0.2 (0.1–0.2)	0.4 (0.3–0.4)	0.4 (0.4–0.5)	< 0.001

*Abbreviations*: *CI* Confidence interval, *HI* Hospital-acquired infection, *PPS* Point prevalence survey

^*^Proportion among all 4367 prescriptions for treatment of hospital-acquired infections

^**^p-values pertain to prescriptions per 100 patients. P-values were calculated with Chi-square test

Over time, the use of penicillins with beta-lactamase inhibitors increased significantly, with piperacillin with beta-lactamase inhibitor accounting for the majority of prescriptions in this group (976 of 1 383, 70.6%; PPS 2022). Moreover, the use of carbapenems increased significantly as well, whereas significantly fewer fluoroquinolones and second-generation cephalosporins were prescribed for treatment of HI.

**Fig. 1:**
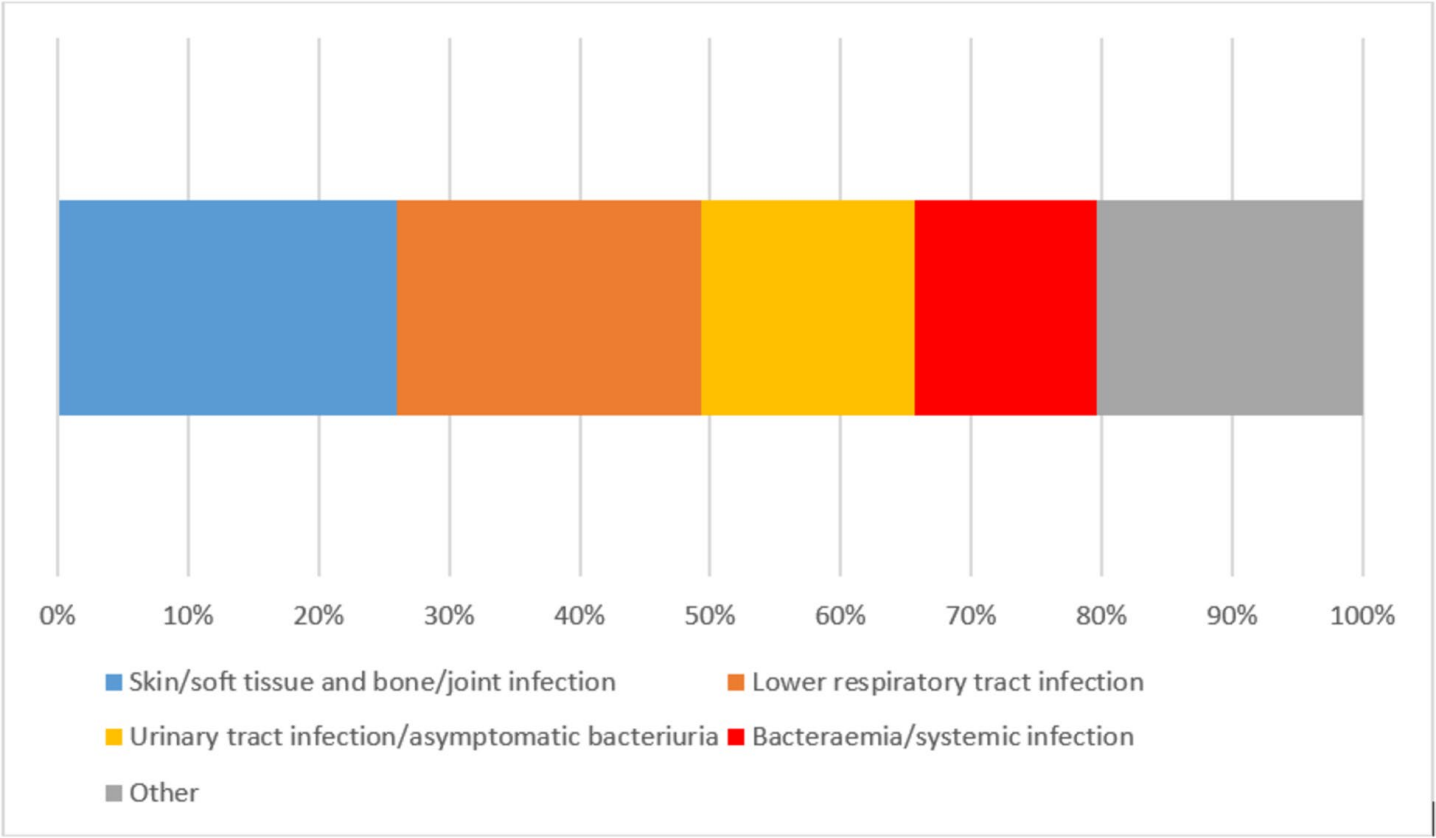
Prescriptions of antimicrobials for the treatment of hospital-acquired infections by site of infection. Data from 252 hospitals participating in the point prevalence survey 2022. Figure legend: Skin/soft tissue and bone/joint infection comprises the following sites of infection: Surgical site infection involving skin or soft tissue but not bone (SST-SSI), Cellulitis, wound, deep soft tissue not involving bone, not related to surgery (SST-O), Septic arthritis, osteomyelitis of surgical site (BJ-SSI), Septic arthritis, osteomyelitis, not related to surgery (BJ-O). Lower respiratory tract infection comprises the following sites of infection: Acute bronchitis or exacerbations of chronic bronchitis (BRON), Pneumonia (PNEU). Urinary tract infection/asymptomatic bacteriuria comprises the following sites of infection: Symptomatic lower urinary tract infection (e.g. cystitis) (CYS), Symptomatic upper urinary tract infection (e.g. pyelonephritis) (PYE), Asymptomatic bacteriuria (ASB). Bacteraemia/systemic infection comprises the following sites of infection: Laboratory-confirmed bacteraemia (BAC), Clinical sepsis (suspected bloodstream infection without laboratory confirmation/results are not available, no blood cultures collected or negative blood culture), excluding febrile neutropenia (CSEP), Systemic inflammatory response with no clear anatomical site (SIRS). Other infections include: Infections of the central nervous system (CNS), Endophthalmitis (EYE), Infections of ear, nose, throat, larynx and mouth (ENT), Cystic fibrosis (CF), Cardiovascular infections: endocarditis, vascular graft (CVS), Gastrointestinal infections (e.g. salmonellosis, antibiotic-associated diarrhoea) (GI), Intra-abdominal sepsis, including hepatobiliary (IA), Obstetric or gynaecological infections, STD in women (OBGY), Prostatitis, epididymo-orchitis, STD in men (GUM), Febrile neutropenia or other form of manifestation of infection in immunocompromised host (e.g. HIV, chemotherapy, etc.) with no clear anatomical site (FN), Completely undefined; site with no systemic inflammation (UND)

The most frequent sites of infection among all HI were skin and soft tissue as well as bone and joint infections (*n* = 1 135, 26.0%), lower respiratory tract infections (bronchitis and pneumonia) (*n* = 1 020, 23.4%) and urinary tract infections/asymptomatic bacteriuria (*n* = 711, 16.3%) (Fig. 1).

When applying the WHO AWaRe classification to both all recorded antimicrobial prescriptions and to prescriptions for treatment of HI specifically, it is noteworthy that the proportion of *Access* antimicrobials was lower for the treatment of HI when compared all indications (24.1% vs. 35.7%, PPS 2022), while the proportion of *Watch* and *Reserve* antimicrobials was higher (62.1% vs. 54.6%, 7.1% vs. 3.1%, PPS 2022). Over time, the prescriptions of a particular AWaRe group per 100 patients remained rather stable (Table 3).

**Table 3:** Antimicrobials according to the WHO AWaRe classification for all indications and specifically for the treatment of hospital-acquired infections. Data from 252 hospitals participating in the point prevalence survey (PPS) 2022, 218 hospitals participating in the PPS 2016 and 132 hospitals participating in the PPS 2011

Indication	AWaRe group	Number of prescription (PPS 2022)	Proportion* (%) (PPS 2022)	Point estimate of prescriptions per 100 patients (95% CI) (PPS 2022)	Point estimate of prescriptions per 100 patients (95% CI) (PPS 2016)	Point estimate of prescriptions per 100 patients (95% CI) (PPS 2011)	*p*-value**
All indications	Access	8002	35.7	12.0 (11.8–12.3)	11.4 (11.1–11.6)	11.2 (10.9–11.5)	< 0.001
	Watch	12,242	54.6	18.4 (18.1–18.7)	20.9 (20.5–21.2)	20.2 (19.8–20.6)	< 0.001
	Reserve	691	3.1	1.0 (1.0–1.1)	0.7 (0.6–0.7)	0.6 (0.5–0.6)	< 0.001
	Not applicable	1487	6.6	2.2 (2.1–2.3)	1.4 (1.3–1.5)	1.9 (1.8–2.1)	< 0.001
Treatment of HI	Access	1052	24.1	1.6 (1.5–1.7)	1.7 (1.6–1.8)	1.6 (1.5–1.8)	0.338
	Watch	2713	62.1	4.1 (3.9–4.2)	4.5 (4.4–4.7)	4.1 (3.9–4.3)	< 0.001
	Reserve	312	7.1	0.5 (0.4–0.5)	0.3 (0.3–0.3)	0.3 (0.2–0.3)	< 0.001
	Not applicable	290	6.6	0.4 (0.4–0.5)	0.3 (0.3–0.4)	0.4 (0.3–0.5)	0.003

Not applicable: Antimicrobials not included in the AWaRe classification

*Abbreviations*: *CI* Confidence interval, *HI* Hospital-acquired infection, *PPS* Point prevalence survey

^*^Proportion among all prescriptions (*n* = 22,422 for all indications; *n* = 4367 for treatment of hospital-acquired infections)

^**^p-values pertain to prescriptions per 100 patients. *P*-values were calculated with Chi-square test

When considering the largest AWaRe group (*Watch*), the strongest increase over time was seen for piperacillin with beta-lactamase inhibitors (1.4 prescriptions per 100 patients in 2011 to 5.9 prescriptions per 100 patients in 2022), while the strongest decrease was observed for ciprofloxacin (3.3 prescriptions per 100 patients in 2011 to 0.8 prescriptions per 100 patients in 2022).

When comparing the distribution of antimicrobial prescriptions of the different AWaRe groups for the respective sites of infection, similar patterns can be observed. *Watch* antimicrobials represented the largest proportion for all sites of infection and were particularly high for the treatment of hospital-acquired lower respiratory tract infections (75.6%). Correspondingly, *Access* antimicrobials were lowest for lower respiratory tract infections (15.4%) (Table 4).

**Table 4:** Antimicrobials according to the WHO AWaRe classification for the treatment of hospital-acquired infection per site of infection. Data from 252 hospitals participating in the point prevalence survey (PPS) 2022

Site of infection	AWaRe group	Number of prescriptions (PPS 2022)	Proportion* (%) (PPS 2022)	Point estimate of prescriptions per 100 patients (95% CI) (PPS 2022)
SST/BJ	Access	385	33.9	0.6 (0.5–0.6)
	Watch	576	50.7	0.9 (0.8–0.9)
	Reserve	106	9.3	0.2 (0.1–0.2)
	Not applicable	68	6.0	0.1 (0.1–0.1)
LRTI	Access	157	15.4	0.2 (0.2–0.3)
	Watch	771	75.6	1.2 (1.1–1.2)
	Reserve	37	3.6	0.1 (0.0–0.1)
	Not applicable	55	5.4	0.1 (0.1–0.1)
UTI/ASB	Access	187	26.3	0.3 (0.2–0.3)
	Watch	463	65.1	0.7 (0.6–0.8)
	Reserve	26	3.7	0.0 (0.0–0.1)
	Not applicable	35	4.9	0.1 (0.0–0.1)
BAC/SYS	Access	155	25.4	0.2 (0.2–0.3)
	Watch	341	55.9	0.5 (0.5–0.6)
	Reserve	71	11.6	0.1 (0.1–0.1)
	Not applicable	43	7.0	0.1 (0.0–0.1)

Not applicable: Antimicrobials not included in the AWaRe classification

*Abbreviations*: *BAC/SYS* Bacteraemia/systemic infection, *CI* Confidence interval, *LRTI* Lower respiratory tract infection, *PPS* Point prevalence survey, *SST/BJ* skin/soft tissue and bone/joint infection, *UTI/ASB* Urinary tract infection/asymptomatic bacteriuria

^*^Proportion among all prescriptions for treatment of hospital-acquired SST/BJ (*n* = 1135), LRTI (*n* = 1020), UTI/ASB (*n* = 711), BAC/SYS (*n* = 610)

## Discussion

This analysis of AU for treating HI in German hospitals demonstrates the interplay between AMS and IPC. The proportion of AU attributed to HI treatment over the past decade, consistently about one-fifth of all antimicrobial prescriptions, reveals that HI are a major driver of AU. The majority of treatment for HI was with broad-spectrum antimicrobials, which suggests that HI impact not only the overall volume of AU, but specifically drive reliance on broad-spectrum options. The high prevalence of broad-spectrum use in Germany aligns with the European average observed in the ECDC PPS 2022 [14]. Our findings underscore an important AMS strategy: reinforcing HI prevention to reduce both AU overall and prescriptions of broad-spectrum agents.

Using the WHO AWaRe classification, we found that *Watch* category antimicrobials constituted a considerable share of AU, and over half of all HI treatment prescriptions. This elevated *Watch* use is consistent with PPS data from other countries [15], and indicates the need to develop policies to transition treatments from broad- to narrower-spectrum agents where clinically feasible. This approach could reduce selective pressure on bacterial populations and potentially lower AMR rates [16, 17]. The observed low use of *Access* antimicrobials (around 36% for all indications) is considerably lower than the EU objective of at least 65% of AU being from this group [18]. Unsurprisingly, the share of *Access* antimicrobials was even lower in AU for the treatment of HI (24%). However, it is important to acknowledge that guidelines for healthcare-associated infections frequently recommend *Watch* antibiotics, for instance, for hospital-acquired pneumonia with risk factors for multidrug resistance or severe courses [19, 20]. This partly explains the high proportion of *Watch* prescriptions. Additionally, the EU target of 65% pertains to total AU and not exclusively to treatment for HI, which should be considered when interpreting our findings.

Our analyses also detail the most common HI sites: skin and soft tissue as well as bone and joint infections, lower respiratory tract infections, and urinary tract infections. Among these, lower respiratory tract infections had particularly high use of *Watch* antimicrobials, which accounted for over 75% of all prescriptions in this category. This pattern is unsurprising, given that lower respiratory tract infections often demand broad-spectrum empiric treatment due to the serious, often polymicrobial nature of these infections [19–21]. However, it also represents an opportunity for AMS to refine diagnostic practices. Enhanced rapid diagnostics could facilitate earlier pathogen-specific treatment, allowing AMS to encourage narrower-spectrum use when appropriate. Notably, we did not capture underlying pathogens, multidrug resistance risk factors or severity levels of the treated infections, making it difficult to assess whether the high *Watch* antimicrobials use was justified.

Comparisons to other studies indicate that the pattern of AU in HI treatment observed in the German PPS is not an isolated phenomenon, as extensive use of *Watch* and *Reserve* antimicrobials is frequently reported across European hospitals [1, 22]. Data from the network for antimicrobial consumption in the EU/EEA (ESAC-Net) confirms this impression. Here, the overall percentage of *Reserve* antimicrobials consumed in the hospital sector in the EU/EEA showed an increasing trend and was reported to be around 5% in the year 2022 [23]. These findings highlight the need for country-specific AMS initiatives, and treatment recommendations that are adapted to local resistance patterns. Improvements in IPC-related metrics, such as increased alcohol-based hand rub consumption and better staffing with IPC personnel, were seen in hospitals participating in the German PPS. However, with regard to AMS staffing that was not the case. Another key observation from this analysis is the relatively low implementation of post-prescription review programmes in German hospitals. The data revealed that, while there has been an increase since 2016, only around a quarter of hospitals reported having a consistent post-prescription review process for antimicrobials across wards. This lag in post-prescription review adoption may contribute to prolonged broad-spectrum use in HI cases, where a more structured review could facilitate timely de-escalation of therapy. Evidence from previous studies suggests that post-prescription reviews are among the most effective AMS interventions, enabling clinicians to optimise therapy based on updated clinical data, including microbiological results [24–26]. AMS is resource-intensive, but currently hardly addressed in medical education in Germany [27, 28]. Results from the PPS fit to perceived deficits and show that AMS is under-implemented in many German hospitals. Potential improvements might include advancing hospital digitalisation through measures such as implementing electronic prescribing systems, patient-level antimicrobial use surveillance, and digital analytical tools [29, 30].

This investigation has several limitations. Participation was voluntary, meaning that selection bias might limit representativeness, especially among smaller or specialised hospitals. When taking the number of acute care hospitals listed in the German hospital registry (*n* = 1 823) as a reference [31], any extrapolations to the national level based on the results of this study, must be made with caution. Although training was provided for data collectors, variability in adherence to PPS protocols across institutions may have introduced inconsistencies. Comparisons over time are also challenging, as participating hospitals differed across PPS iterations (93 hospitals participated in both the PPS 2016 and the PPS 2022, with 25 hospitals participating in all three surveys). The light version of the PPS protocol was adopted in Germany, which, while facilitating broad data collection, may lack certain clinical and contextual details. Furthermore, prescription for treatment of HI reflects the assessment of the treating physicians, and was not validated against ECDC criteria. Accordingly, no information was obtained regarding causative microorganisms for treated HI, rendering an assessment of the appropriateness of recorded antimicrobial treatments difficult. While the AWaRe classification offers a useful framework, its applicability in the acute care hospital setting, where patient acuity and clinical complexities often necessitate broad-spectrum AU, requires further investigation. Lastly, the descriptive nature of this investigation limits causal inferences and longitudinal studies may provide deeper insights into factors affecting AU trends.

The results of this investigation provide a valuable snapshot of AU in the treatment of HI in German hospitals. Repeating the PPS at regular intervals will allow for consistent tracking of AU and stewardship practices. It will be important to remain mindful that the WHO AWaRe classification may evolve over time, which could impact future comparisons and assessments.

## Conclusion

The results of the German PPS 2022 underscore that AU for HI treatment remains substantial in German hospitals, with a high reliance on *Watch* category antimicrobials despite AMS and IPC advancements. The findings highlight the critical role of IPC improvements in reducing AU, particularly with broad-spectrum agents. Adoption of post-prescription review policies may represent a promising, underutilised resource for AMS to encourage de-escalation when appropriate. By strengthening AMS and IPC in tandem, German hospitals will be better equipped to address AMR.

## Data Availability

Not applicable, because all data were collected within the context of the surveillance of healthcare-associated infections conducted in accordance with the German Protection against Infection Act.

## Abbreviations

AMS: Antimicrobial stewardship
AU: Antimicrobial use
AWaRe: Access, Watch, Reserve
ECDC: European Centre for Disease Prevention and Control
HI: Hospital-acquired infection
IPC: Infection prevention and control
KISS: Krankenhaus-Infektions-Surveillance-System
PPS: Point prevalence survey
